# First report of multiple lineages of dengue viruses type 1 in Rio de Janeiro, Brazil

**DOI:** 10.1186/1743-422X-8-387

**Published:** 2011-08-03

**Authors:** Flavia B dos Santos, Fernanda B Nogueira, Márcia G Castro, Priscila CG Nunes, Ana Maria B de Filippis, Nieli RC Faria, Jaqueline BS Simões, Simone A Sampaio, Clarice R Santos, Rita Maria R Nogueira

**Affiliations:** 1 Laboratório de Flavivirus, Oswaldo Cruz Institute, Rio de Janeiro, Brazil; 2 Laboratório de Transmissores de Hematozoários, Oswaldo Cruz Institute, Rio de Janeiro, Brazil

**Keywords:** Dengue virus type 1, multiple lineages, phylogeny, Rio de Janeiro

## Abstract

**Background:**

In Brazil dengue has been a major public health problem since DENV-1 introduction and spread in 1986. After a low or silent co-circulation, DENV-1 re-emerged in 2009 causing a major epidemic in the country in 2010 and 2011. In this study, the phylogeny of DENV-1 strains isolated in RJ after its first introduction in 1986 and after its emergence in 2009 and 2010 was performed in order to document possible evolutionary patterns or introductions in a re-emergent virus.

**Findings:**

The analysis of the E gene sequences demonstrated that DENV-1 isolated during 2009/2010 still belong to genotype V (Americas/Africa) but grouping in a distinct clade (lineage II) of that represented by earlier DENV-1 (lineage I). However, strains isolated in 2011 grouped together forming another distinct clade (lineage III).

**Conclusions:**

The monitoring of DENV is important to observe the spread of potentially virulent strains as well to evaluate its impact over the population during an outbreak. Whether explosive epidemics reported in Brazil caused mainly by DENV-1 was due to lineage replacement, or due the population susceptibility to this serotype which has not circulated for almost a decade or even due to the occurrence of secondary infections in a hyperendemic country, is not clear. This is the first report of multiple lineages of DENV-1 detected in Brazil.

## Findings

Dengue viruses (DENV) are the most important human arboviruses worldwide, transmitted by mosquitoes of the genus *Aedes *and currently it is estimated that 70 to 500 million dengue infections occur annually in 124 endemic countries. Nearly 3.6 billion people (55% of world population) are at risk of contracting the disease [1]. The rapid global spread of the four DENV serotypes (DENV-1 to 4) in the last 50 years resulted in the dispersal of genotypes associated with increased severity [2].

**Figure 1:**
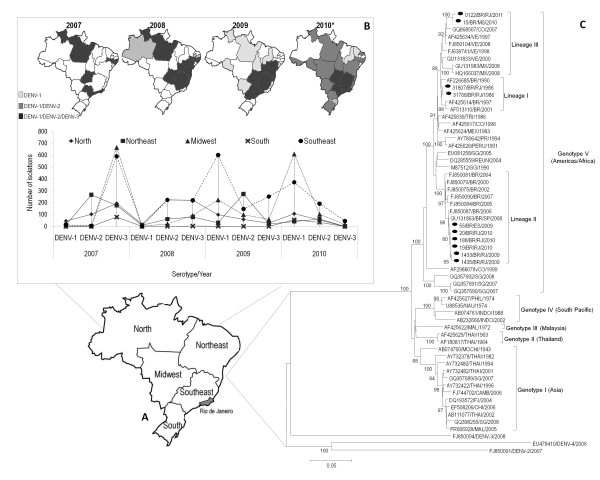
**DENV-1 re-emergence in Brazil**. (A): Brazil's five geographical regions: North, Northeast, Midwest, Southeast and South. In gray, Rio de Janeiro's localization. (B): Dengue viruses (DENV) serotypes replacements and DENV-1 emergence in Brazil, by region from 2007 to 2010. (C): Neighbor-joining phylogenetic of ten complete envelope (E) gene sequences from DENV-1 isolated during two periods epidemiologically distinct: 1986 (*n = 2*) when the serotype was first introduced and after its emergence in 2009-2011 (*n = 8*). The percentage of replicate trees in which the associated taxa clustered together in the bootstrap test (1000 replicates) is shown next to the branches. Black circles represent DENV-1 sequences generated in this study. DENV strains used were named as follows: GenBank accession number/country/year.

In Brazil, the State of Rio de Janeiro (RJ), in the Southeast region (Figure 1A) has been important to the epidemiology of dengue, with the introduction of DENV-1 in 1986, DENV-2 in 1990 and DENV-3 in 2000 [3]. The latter was prevalent in the majority of Brazilian States from 2002 to 2006 and, from 2007 to 2009 this serotype was displaced by DENV-2. In 2008, the Southeast and the Northeast regions were responsible for approximately 80% of the cases reported in the most severe epidemic in the country, where DENV-2 and DENV-3 were detected in 96.4% of the cases isolated. After a low or silent circulation, DENV-1 re-emerged in the Southeast region in 2009 (Figure 1B) and it was the serotype detected in 50.4% of the viral isolations, displacing DENV-2 (30.5%) and DENV-3 (19.1%) [4].

DENV-1 falls into five distinct genotypes designated as genotype I (Southeast Asia, China and East Africa), genotype II (Thailand), genotype III (Malaysia), genotype IV (South Pacific) and genotype V (America/Africa) and the existence of lineages with distinct geographic and temporal relationships have been suggested [5,6]. Moreover, lineage turnover or replacements have become more frequently common in phylogenetic studies. The term "lineage" has been used non- officially to characterize those viruses clustered in clades in a taxonomic level beneath genotype [7].

In this study, the phylogeny of DENV-1 strains isolated in RJ after its first introduction in 1986 and after its emergence in 2009 and 2010 was performed in order to document possible evolutionary patterns or introductions in a re-emergent virus. Phylogenetic studies may constitute an important tool to monitor the introduction and spread of viruses as well as to predict the potential epidemiological consequences of such events.

The strains analyzed in this study belong to a collection obtained from acute phase human serum through the passive surveillance system performed by the Laboratory of Flavivirus, IOC/FIOCRUZ, Rio de Janeiro, Brazil, from an ongoing Project approved by resolution number CSN196/96 from the Oswaldo Cruz Foundation Ethical Committee in Research (CEP 274/05), Ministry of Health-Brazil. To avoid mutations introduced by *in vitro *passages of the virus in cell cultures we used DENV-1 strains (*n = *10; from 1986 [n = 2], 2009 [n = 3], 2010 [n = 4] and 2011 [*n = *1]) extracted directly from serum previously detected by RT-PCR or originally isolated from cell culture when serum did not yield enough volume for RNA extraction.

DENV-1 isolation was performed by inoculation into C6/36 *Aedes albopictus *cell line [8] and isolates were identified by indirect fluorescent antibody test (IFAT) using serotype-specific monoclonal antibodies [9]. RT--PCR for detecting and typing DENV from serum was performed as described previously [10]. For RT-PCR and sequencing the viral RNA was extracted from infected cell culture supernatant or directly from the patients serum using QIAamp Viral RNA Mini kit (Qiagen) following the manufacturer's instructions and stored at -70°C for DENV typing and sequencing.

The sequencing reaction was performed by reverse transcription using 5 μL of extracted RNA in 25 μL of AccessQuick™ RT-PCR System (Promega Corporation) and specific oligonucleotides primers which sequences can be provided upon request, to amplify the C/prM/M/E region of 2,325 bp. Amplification was conducted using a Model 9700 thermal cycler (Applied Biosystems). PCR products were purified from using QIAquick Gel extraction Kit or QIAquick PCR purification Kit (Qiagen) and used as template for cycle sequencing. Sequencing reactions were performed as recommended in the BigDye Dideoxy Terminator sequencing kit (Applied Biosystems) and the products were analyzed using an automated 3130 DNA Sequencer (Applied Biosystems). Sequences for the complete E gene (1,485 nucleotides) were deposited in GenBank (http://www.ncbi.nlm.nih.gov).

The sequences multiple alignment was performed using CLUSTAL W (http://www.ebi.ac.uk/clustalw/) and the phylogenetic analysis by MEGA 4 software (http://www.megasoftware.net), using the "Neighbor-joining" method, according to the Tamura-Nei model, with a bootstrap of 1,000 replications. Strains representative from the five genotypes available in Genbank (http://www.ncbi.nlm.nih.gov) were used for the comparison, DENV-2, DENV-3 and DENV-4 strains were used as outgroup to root the trees.

The results based on the analysis of the E gene sequences have demonstrated that the DENV-1 strains isolated during 2009/2010 in RJ and one isolated in the State of Espirito Santo (ES) used for comparison purposes, still belong to genotype V (America/Africa) previously detected in the country, but grouping into a distinct clade (lineage II) of that represented by earlier Brazilian DENV-1 strains (lineage I) with a strong bootstrap support. In fact, the re-emergent DENV-1 was more closely related to strains isolated in Singapore in 1990 and in 2005, suggesting a probable Asian origin. However, one strain isolated in 2010 (15/BR/MS/2010) from a RJ resident who traveled to Mato Grosso do Sul (MS), Midwest region and one strain recently isolated in 2011 in RJ (0122/BR/RJ/2011) grouped together forming another distinct clade (lineage III), grouping with strains isolated in 2007 and 2008 in Colombia, Venezuela and Mexico, suggesting a Latin American origin for those strains (Figure 1C).

In spite of the continuous low circulation in the country, the low percentage of identity of the newly isolated viruses with those strains first introduced in the 80's suggest that the re-emergent DENV-1 did not evolved locally but occurred probably due to new lineages introductions in the country (Table 1). The analysis based on the E gene sequences from DENV-1 strains isolated in the Northern region of Brazil from 2000 to 2008 available on GenBank support the idea that those viruses could have been introduced earlier and their low or silent circulation could be due to the prevalent DENV-3/DENV-2 circulation during that time (unpublished data). The circulation of more than one DENV-1 "lineage" has been described in Asia [6] and in the Americas [7]. A previous study by Carrillo-Valenzo [11] recently reported multiple viral lineages introductions for each DENV serotype in Mexico with frequent lineage replacements. In fact, lineage replacements appear to be a more common observation than long term lineage persistence [12].

**Table 1:** Sequences identity between Brazilian DENV-1 based on the E gene analysis (1,485 nucleotides)

DENV-1 Strains	55/2009^a^	1435/2009	1433/2009	15/2010	19/2010	20/2010	188/2010	0122/2011	31768/1986	31807/1986
**55/2009**	-	99,6^b^	99,6	95,8	99,8	99,8	99,8	95,6	96,8	96,9
**1435/2009**	**99,7**	-	100,0	95,6	99,7	99,7	99,7	95,4	96,6	96,7
**1433/2009**	**99,7**	**100**	-	95,6	99,7	99,7	99,7	95,4	96,6	96,7
**15/2010**	**99,1**	**98,9**	**98,9**	-	95,8	95,8	95,8	99,4	98,1	98,1
**19/2010**	**100**	**99,7**	**99,7**	**99,1**	-	100	100	95,6	96,8	96,9
**20/2010**	**100**	**99,7**	**99,7**	**99,1**	**100**	-	100	95,6	96,8	96,9
**188/2010**	**100**	**99,7**	**99,7**	**99,1**	**100**	**100**	-	95,6	96,8	96,9
**0122/2011**	**98,9**	**98,9**	**98,9**	**99,7**	**98,9**	**98,9**	**98,9%**	-	97,7	97,7
**31768/1986**	**99,1**	**98,9**	**98,9**	**99,5**	**99,1**	**99,1**	**99,1%**	**99,3**	-	99,6
**31807/1986**	**98,9**	**98,7**	**98,7**	**99,3**	**98,9**	**98,9**	**98,9%**	**99,1**	**99,7**	-

^a^: Brazilian strains analyzed in this study. Strain name followed by year of isolation; ^b^: percentage of nucleotide identity as determined by BioEdit (http://www.mbio.ncsu.edu/bioedit/bioedit.html); ^c^: percentage of amino acid identity (bold).

Lineage replacement occurs when an entire clade of viruses that has persisted in a particular locality for a period of time is not evident on a subsequent sampling, indicating that it has dropped dramatically in frequency, even experiencing extinction, and sometimes replaced by a new clade of viruses [13]. Despite this, the evolutionary processes controlling these events are not fully understood. Recently, it has been suggested that despite the endemicity of a particular serotype in a specific geographic region for a long period, different viral clades may be involved in that period [14]. It is known that the introduction of new DENV serotypes/genotypes/lineages is a major risk factor for dengue epidemics. In 2009/2010, 1,471,390 dengue fever (DF) suspected cases and 665 deaths were reported in Brazil, with DENV-1 causing epidemics in most states [4,15]

It is not clear whether the explosive epidemic reported in Brazil during 2009 and 2010 caused mainly by DENV-1 was due to this lineage replacement. The population susceptibility to this serotype which has not circulated for almost a decade and the occurrence of secondary infections in a hyperendemic country may also have played an important role in the disease epidemiology,. In this scenario, the monitoring of DENV is of great relevance to observe the spread of potentially virulent strains as well to evaluate its impact over the population during an outbreak.

Due to the Brazil's geography and dengue epidemiology, along with the fact the country has important tourist regions, a larger sampling analysis is suggested to better characterize those replacement events and lineage introductions in the country.

## Competing interests

The authors declare that they have no competing interests.

## Authors' contributions

FBS, RMRN and AMBF designed the study, FBN, NRCF, SAS, JBSS and CS performed the experiments, NCF and PCGN analyzed the data. FBS and FBN wrote the paper. All authors have read and approved the final manuscript.

## Funding

This work was supported by CNPq [303546/2008-5], CAPES, FAPERJ [E/26-102.936/2008] and FIOCRUZ.
